# Sensor technologies for the detection and monitoring of endocrine-disrupting chemicals

**DOI:** 10.3389/fbioe.2023.1141523

**Published:** 2023-03-27

**Authors:** Muhammad Musaddiq Shah, Khurshid Ahmad, Sonia Boota, Tor Jensen, Michael R. La Frano, Joseph Irudayaraj

**Affiliations:** ^1^ Department of Biological Sciences, Faculty of Sciences, University of Sialkot, Sialkot, Pakistan; ^2^ College of Food Sciences and Engineering, Ocean University of China, Qingdao, Shandong, China; ^3^ Biomedical Research Center, Mills Breast Cancer Institute, Carle Foundation Hospital, Urbana, IL, United States; ^4^ Metabolomics Core Facility, Roy J Carver Biotechnology Center, The University of Illinois at Urbana-Champaign, Urbana, IL, United States; ^5^ Department of Bioengineering, The University of Illinois at Urbana-Champaign, Urbana, IL, United States; ^6^ Micro and Nanotechnology Laboratory, The University of Illinois at Urbana-Champaign, Urbana, IL, United States

**Keywords:** endocrine-disrupting chemicals, environmental contaminants detection, analytical technics, biosensors, human health and disease

## Abstract

Endocrine-disrupting chemicals (EDCs) are a class of man-made substances with potential to disrupt the standard function of the endocrine system. These EDCs include phthalates, perchlorates, phenols, some heavy metals, furans, dimethoate, aromatic hydrocarbons, some pesticides, and per- and polyfluoroalkyl substances (PFAS). EDCs are widespread in the environment given their frequent use in daily life. Their production, usage, and consumption have increased many-fold in recent years. Their ability to interact and mimic normal endocrine functions makes them a potential threat to human health, aquatics, and wild life. Detection of these toxins has predominantly been done by mass spectroscopy and/or chromatography-based methods and to a lesser extent by advanced sensing approaches such as electrochemical and/or colorimetric methods. Instrument-based analytical techniques are often not amenable for onsite detection due to the lab-based nature of these detecting systems. Alternatively, analytical approaches based on sensor/biosensor techniques are more attractive because they are rapid, portable, equally sensitive, and eco-friendly. Advanced sensing systems have been adopted to detect a range of EDCs in the environment and food production systems. This review will focus on advances and developments in portable sensing techniques for EDCs, encompassing electrochemical, colorimetric, optical, aptamer-based, and microbial sensing approaches. We have also delineated the advantages and limitations of some of these sensing techniques and discussed future developments in sensor technology for the environmental sensing of EDCs.

## 1 Introduction

Due to rapid industrial advances, hundreds of new chemicals have been synthesized for domestic and industrial use without a clear understanding of the toxic effects on humans and the long-term deleterious effects on the ecosystem (Ren et al., 2015b; Esteso, 2022). EDCs are a group of structurally diverse, artificially synthesized exogenous chemicals with the ability to perturb the normal function, secretion, transport, and metabolism of natural hormones (Chen H. et al., 2019; Charitos et al., 2022). These hormones are produced by the endocrine glands (hypothalamus, pituitary, thyroid, parathyroid, thymus, and adrenal), have many regulatory effects on natural homeostasis, and affect the development and overall function of the endocrine system. EDCs can enter the body through several routes such as inhalation, skin contact, and ingestion to affect the natural functions of the endocrine hormones (Roy et al., 1997; Bergman et al., 2013; Preciados et al., 2016). Several EDC compounds are found contemporaneously in pesticides, insecticides, pharmaceutical agents, compounds of heavy metals, drugs, feed additives, industrial chemicals, and electronic waste (e-waste). Because of the impact of the endocrine system on healthy development and regulation of hormones, EDCs have a devastating potential to adversely affect individuals of every group from childhood to old age (Lee et al., 2015; Preciados et al., 2016).

EDCs are present in a wide range of synthetic plastic products such as toys, bottles, and PVC pipes. They are also commonly found in detergents, toothpaste, flame retardants, and in different cosmetic items (Flint et al., 2012). EDCs as pesticides are extensively applied in the agriculture industry for the improvements of crop yields. The residues of these toxins are detected in diverse food products and their related environments (Yang et al., 2020). For example, Atrazine is detected in surface and groundwater all over the world (Wan et al., 2021). EDCs enter the environment *via* wastewater discharge, hospital waste disposal, leaching of chemicals from industrial plants and households, and pesticide residues. They often enter landfills and reach aquatic bodies, such as surface water, groundwater (Gao et al., 2020), the marine environment (Muller et al., 2020), freshwater reservoirs (Rocha et al., 2019), and ultimately enter the food chain through sea food contamination (Muller et al., 2020; Chen et al., 2022; Shah et al., 2022).

Per- and poly-fluoroalkyl substances (PFAS) which have been brought to the limelight are ubiquitous and reported to be present in surfactants, lubricants, fire retardants, and polymer additives (Hoisaeter et al., 2019; Mumtaz et al., 2019; Xu et al., 2021). Dichlorodiphenyltrichloroethane (DDT) is an insecticide commonly used to combat malaria (Meeker, 2012). Polychlorinated biphenyls (PCBs) have been extensively used in electrical equipment, mainly in transformers and capacitors (Habibullah-Al-Mamun et al., 2019). Phthalates are extensively used in children’s products, medical devices, cosmetics goods, plastic items, packing, and building materials (Vieira et al., 2021). Bisphenol A, an estrogen mimic, is widely used in the plastics industry and in food packaging, thermal papers, DVDs, plastic pipes, vehicle panels, medical equipment, personal care products, and dental sealants (Fisher et al., 2017). It has also been used as a supplement in cattle and poultry production to increase growth rates (Pironti et al., 2021). Cadmium is used in the production of batteries, pigments, plastic, and alloys (Friberg et al., 2019). The use of Lead is reported in plastic, glass, batteries, and welding materials. Mercury is used in the production of pigments, caustic soda, thermometers, and in dental products. As a result of extensive uses and applications of these toxins, EDCs are widespread in our ecosystem contributing to conditions such as diabetes, obesity, nervous disorders, gland dysfunction, discomfort in respiration, abnormalities in muscular functions, seizures, cardiovascular diseases, fluctuation in blood pressure, early puberty, and infertility (Vieira et al., 2021).

Many EDCs have the ability to mimic the functions of natural hormones, impacting many physiological functions such as growth, development, and reproduction through the overstimulation of receptors (Lu et al., 2020). Other EDCs block the activity of natural hormones, disrupting normal signaling through the inhibition of binding. This leads to the disturbance in the function of glands, abnormal levels of hormone production, secretions, and metabolism, which ultimately lead to adverse health outcomes for the exposed individual (Vandenberg et al., 2012; Hu et al., 2015; Vandenberg, 2019). Exposure to EDCs leads to many diseases which are caused by a disturbance in the normal functionality of estrogen, androgen, and thyroid hormones. Structural similarities of EDCs provide the potential to interact with nuclear and hormonal receptors, resulting in the activation of hormone transporter proteins, dysregulation of healthy hormone metabolism, and affect the number of receptors on cell surfaces, which lead to the interruption in normal hormonal responses. EDCs affect the signal transduction to the immune as well as the nervous systems and often lead to alteration of the production and function of natural hormones (De Coster and Van Larebeke, 2012). The lipophilic nature of many of these chemicals causes them to be retained in adipose tissues, which leads to prolonged and elevated levels of these chemicals inside living beings (Heindel et al., 2015; Sargis, 2015). EDCs also have negative impacts on genome regulation. Epigenetic changes such as methylation and acetylation of DNA and histone modifications are also being reported (Zama and Uzumcu, 2010; Lauretta et al., 2019). Even a low dose of these chemicals have the potential to either directly or indirectly affect the exposed individuals. Experimental results suggest that the low concentration of these chemicals showing impairment during *in vitro* experiments can also be detrimental not only to wildlife but to human health as well (Lauretta et al., 2019).

Many *in vitro* and *in vivo* studies report the health damages due to exposure to EDCs, including short-term acute and long-term chronic effects, which may appear months or years after exposure (Hayes et al., 2011; Sabuz Vidal et al., 2021; Sakhteman et al., 2021). Acute effects which are observed in persons involved in the use of pesticides include painful eyes, rashes, wounds, blindness, nausea, respiratory issues, and diarrhea (De Coster and Van Larebeke, 2012; Ren et al., 2015a). The chronic effects which are reported include cancer, congenital disabilities, neural and developmental deformation as well as immunotoxicity (Nalbone et al., 2013; Scognamiglio et al., 2016). The brain and nerve cells are very prone to be affected by these EDCs, especially at the developmental stages (Marinello and Patisaul, 2021). Experimental results and clinical and epidemiological studies confirm that exposure to EDCs can result in harmful effects on brain health (Lopez-Rodriguez et al., 2021). The toxic nature of EDCs has the potential to alter normal functions of neural cells that result in neurogenesis and affect neural transmission. This can lead to neurological disorders that can disrupt learning and cause aggressive behavior (Kajta and Wojtowicz, 2013; Preciados et al., 2016; Streifer and Gore, 2021).

Conventional measurement of EDCs has been primarily performed using liquid or gas chromatography-mass spectrometry (LC- or GC-MS) in a variety of matrices such as water, breast milk, urine, serum, food, and soil (Metcalfe et al., 2022). PFAS and phthalates have most commonly been measured by LC-MS using tandem mass spectrometry (MS/MS) (Dima et al., 2020; Abafe et al., 2021) or high resolution mass spectrometry (HRMS) (Charbonnet et al., 2022) In contrast, aromatic hydrocarbons have been primarily measured using GC-MS or GC-HRMS (Wang et al., 2019; Zastrow et al., 2021) while BPA, insecticides, and pesticides have been analyzed using either LC-MS/MS or GC-MS (Lee et al., 2017; Dima et al., 2020). Metal EDCs such as arsenic and lead have most often been tested by inductively coupled plasma mass spectrometry (ICP-MS) (Luo et al., 2017). In general, while targeting specific EDCs using such techniques as MS/MS provides the optimal ability to detect low abundant compounds, HRMS enables the greater ability to discover unknown EDC metabolites through untargeted screening (Metcalfe et al., 2022). Due to the variety of sample matrices and extraction methods used prior to MS analysis, the use of internal standards is critical for ensuring precision and accuracy of quantitation (Cortese et al., 2020). Surrogate internal standards, consisting of stable isotope or deuterium labeled versions of target compounds, are added at the beginning of extraction to later adjust data for factors including extraction efficiency and matrix effects. In some instances, such as with PFAS, internal standard mixtures consisting of a large number of compounds within an EDC class can be purchased to facilitate inclusion in extraction protocols (Genualdi and deJager, 2021).

Monitoring and detection of EDCs compounds, as noted earlier, have been traditionally performed with analytical methods such as gas chromatography-mass spectrometry (GC-MS) (Azzouz et al., 2020), Liquid chromatography (Zatrochova et al., 2022), ultra-performance liquid chromatography-tandem mass spectrometry (Zhou et al., 2022), capillary electrophoresis (Chen et al., 2021) surface plasmon resonance (Bakhshpour and Denizli, 2020), and solid phase extraction (Zhang et al., 2020). Although conventional analytical methods are standardized, highly sensitive and selective, these approaches are time-consuming, have complex protocols, often require sample pre-treatment and sophisticated instrumentation, trained and skilled personnel. Novel sensing systems have been reported and well-established in the last decades to monitor several toxins. Recent advances in materials and detection systems provide rapid, amenable for on-site, real-time, and cost-effective monitoring at very high sensitivity of EDCs at trace levels. Table 1 provides a concise account of technologies used for detection various EDCs.

**TABLE 1:** Instrument-based techniques for monitoring EDCs in selected samples.

	Chemical name	Conventional detection method	Type of sample	LOD (mol L^−1^)	References
1.	Perchlorate	Carbon Nanotubes/Solid-contact ISEs	--	1.8 × 10^−7^	Hassan et al. (2019)
2.	Phthalate	Electrochemical impedance sensing platform	--	4.5 × 10^−9^	Bolat et al. (2019)
		GC-MS	Human Saliva	9.14 × 10^−11^ –1.82 × 10^−9^	Vu et al. (2020)
3.	Bisphenol A	GC-MS	Dairy products	2.62 × 10^−11^–.01 × 10^−10^	Palacios Colon et al. (2021)
			Water	9.14 × 10^−11^–1.82 × 10^−9^	Alimzhanova et al. (2022)
		---	Fish (Muscle)	1.00 × 10^−10^	Petrarca et al. (2022)
		GC-MS	Planaria	--	St. John et al. (2021)
		DLLME- GC-MS/MS	Placenta Tissues	1.75 × 10^−10^–3.50 × 10^−10^	Fernandez et al. (2021)
		GC-MS/MS	Ambient air	3.06 × 10^−10^–6.57 × 10^−10^	Naing et al. (2021)
		HPLC-UV and LC-MS/MS	Whole milk	1.09 × 10^−7^–2.19 × 10^−7^	Mesa et al. (2019)
4.	Heavy Metal Ions	SPR	Water, Aqueous Solutions	8.89 × 10^−11^	Bakhshpour and Denizli (2020)
				0.28 nm × 10^–3^	Dhara et al. (2019)
				0.55 nm × 10^–6^	Chen et al. (2019c)
5.	Organophosphates	CE	Fruit and vegetable juice	4.27 × 10^−8^	Chen et al. (2021)
				1.16 × 10^−10^	Li et al. (2018a)
		LC-MS/MS	Environmental water	7.38 × 10^−11^ and 8.92 × 10^−12^	Cakır and Baysal (2019)
		SPR	Environmental water	3.65 × 10^−12^ and 3.10 × 10^−11^	Cakır and Baysal (2019)
6.	PAHs	GC-MS/MS	Cow milk	1.18 × 10^−9^–3.59 × 10^−9^	Hasan et al. (2022)

The impact of EDCs on health is amplified by both a dearth of knowledge of how these chemicals affect our wellbeing and by a lack of awareness of how pervasive these compounds are in our environment (Kabir et al., 2015). In this review, we will focus on new or emerging technologies for the detection of EDCs. We discuss biosensors based on electrochemical, colorimetric, optical, aptamer, and microbial sensing approaches for EDC detection. The advantages and limitations of different sensing systems are compared and evaluated in the context of more conventional, lab-based detection techniques. Future developments and applications of EDC sensors will be discussed in the last section.

## 2 Sensing systems for EDCs

Sensors are integrated devices that require small sample volumes and minimum sample preparation, are fast, cheap, portable, user-friendly, and capable of performing real-time monitoring without the production of toxic chemicals. While there are several success stories, summarized in Table 2, numerous systems are still in development. In the subsequent sections, we will discuss the different biosensing modalities for the detection of EDCs.

**TABLE 2:** Advanced sensing techniques for the detection of EDCs.

Analyte	Sensor technology	Sensing approach	LOD (mol L^−1^)	Type of sample	Ref.
Atrazine	QD	Fluorescence	0.80 × 10^−7^	Water	Nsibande and Forbes (2019)
	Nitrogen-doped QD	Luminescent probe	3 × 10^−12^	Vegetables	Mohapatra et al. (2018)
	SnO_2_ Nanofibers	Electrochemical	0.9 × 10^−21^	Water	Supraja et al. (2019)
	Carbon Nanotubes	Immunosensor	4.63 × 10^−12^	Water	Belkhamssa et al. (2016)
	MIPs	Electrochemical sensor	8.80 × 10^−8^	Water	Che Lah et al. (2021)
	Capped AgNPs	Electrochemical sensor	1.77 × 10^−7^	--	Zahran et al. (2020)
	MIP-Based	Potentiometric	4 × 10^−7^	Tap water	Alberti et al. (2022)
	NiHCF NPs and reduced graphene oxide.	Electrochemical aptasensor	0.1 × 10^−12^	Water	Fan et al. (2019)
BPA	biochar NPs	Electrochemical	3.18 × 10^−9^	Water	Liu et al. (2019)
	Aptamers Based	Colorimetric detection	2.02 × 10^−9^	Milk, orange juice, and mineralized water	Jia et al. (2020)
	AuNPs	Colorimetric aptasensor	0.004 × 10^−9^	Grain	Lee et al. (2019)
	AuNPs and carbon black Nano composite	Electrochemical sensor	60 × 10^−9^	Water	Jebril et al. (2021)
	Engineered *Escherichia coli* cells	Electrochemical biosensor	0.01 × 10^−9^	Tea and juice samples	Zhao et al. (2022)
	Aptasensor	Ellipsometric method	36 × 10^−12^	--	Sahin et al. (2022)
Perchlor ate	lux biosensors, bioluminescence	Bacterial luciferase *lux* genes	--	Water	Balabanov et al. (2017)
	AuNPs	Colorimetric sensing	2.4 × 10^−5^	Soil	Keskin et al. (2020)
	Pt(II) terpyridyl complex-based sensing	Colorimetric and luminescent dual-mode	0.45 ng (naked eye)	Soil and air	Su et al. (2022)
	AuNPs	Colorimetric Sensor	1.5 × 10^−6^	Sparkler filtrate	Keskin et al. (2022)
	Platinum (II) complex in a hydrogel matrix	Luminescent turn-on	1.12 ng (naked eye)	--	Su et al. (2021)
OP Pesticides	gold nanorods	Multicolor sensor	8.58 × 10^−10^	Food samples	Yin et al. (2021)
	UCNPs- MnO_2_	Aptasensor	2.61 × 10^−10^	--	Ouyang et al. (2021)
	C-AuNPs	Colorimetric detection	1.99 × 10^−8^	Environmental sample	Shah et al. (2021)
	Gold Nanoparticles	Nano Gold- electrochemical biosensor	7.38 × 10^−11^–2.99 × 10^−10^	--	Zhao et al. (2021)
	Cu@Ag Nanoparticles	Colorimetric detection	4.23 × 10^−8^	fruit extract and environmental sample	Faghiri et al. (2021)
	Gold Nanoparticles	Colorimetric detection	1.77 × 10^−7^		Ma et al. (2017)
	--	Fluorometric sensing	4.52 × 10^−10^	--	Liu et al. (2020)
Phthalate esters	ZIF-8 fluorescent MOF NPs	Optical sensing	3.32 × 10^−5^ –9.98 × 10^−5^	--	Chapartegui Arias et al. (2019)
	Quantum dot	aptasensor	<2.56 × 10^−7^	--	Lim et al. (2018)
	AuNPs@β-CD	Colorimetric/SERS sensor	14.9 × 10^−9^	Liquor and rice wine	Li J et al. (2019)
	AuNPs	Electrochemical	2.51 × 10^−8^	Water samples	Liang et al. (2017)
	SiO2@QDs@MIPs	Fluorescence	---	Tap water	Zhou et al. (2017)
	MWCNTs and AuNPs	MIP electrochemical sensor	1.83 × 10^−11^	--	Wang et al. (2022)
Heavy Metals Ions	Gold nanoparticles	Colorimetric sensing	--	Water	Gunupuru et al. (2022)
PFOS	Macrocycle Bowtie Cyclophane	Fluorescence detection	47.3 ± 2.0 ×10^−9^	Water	Lei and Cong (2022)
	QDs	Fluorescent and visual sensor	18.27 × 10^−9^	Water	Chen et al. (2019b)

### 2.1 Electrochemical sensors for the detection of EDCs

An electrochemical sensor is made of two electrodes, i.e., reference and sensing electrodes. A suitable electrolyte separates both electrodes. The presence of a target analyte generates electrochemical signals which are used to monitor the concentration of the analyte (Kaya et al., 2021). These types of sensing systems provide high selectivity, low power requirements, linear and stable output, excellent accuracy, repeatability, and economical sensing. With all these features, the easy integration of these electrochemical sensors into smart portable units makes them a useful option for in-field deployment (Hayat and Marty, 2014). Their compatibility with complicated sample matrices reduces the need for lengthy sample pretreatments and leads to the simplification of sensing protocols (Lee and Irudayaraj, 2013; Huang et al., 2017). All these are attractive features in the development of electrochemical sensors for the detection of EDCs.

**FIGURE 1:**
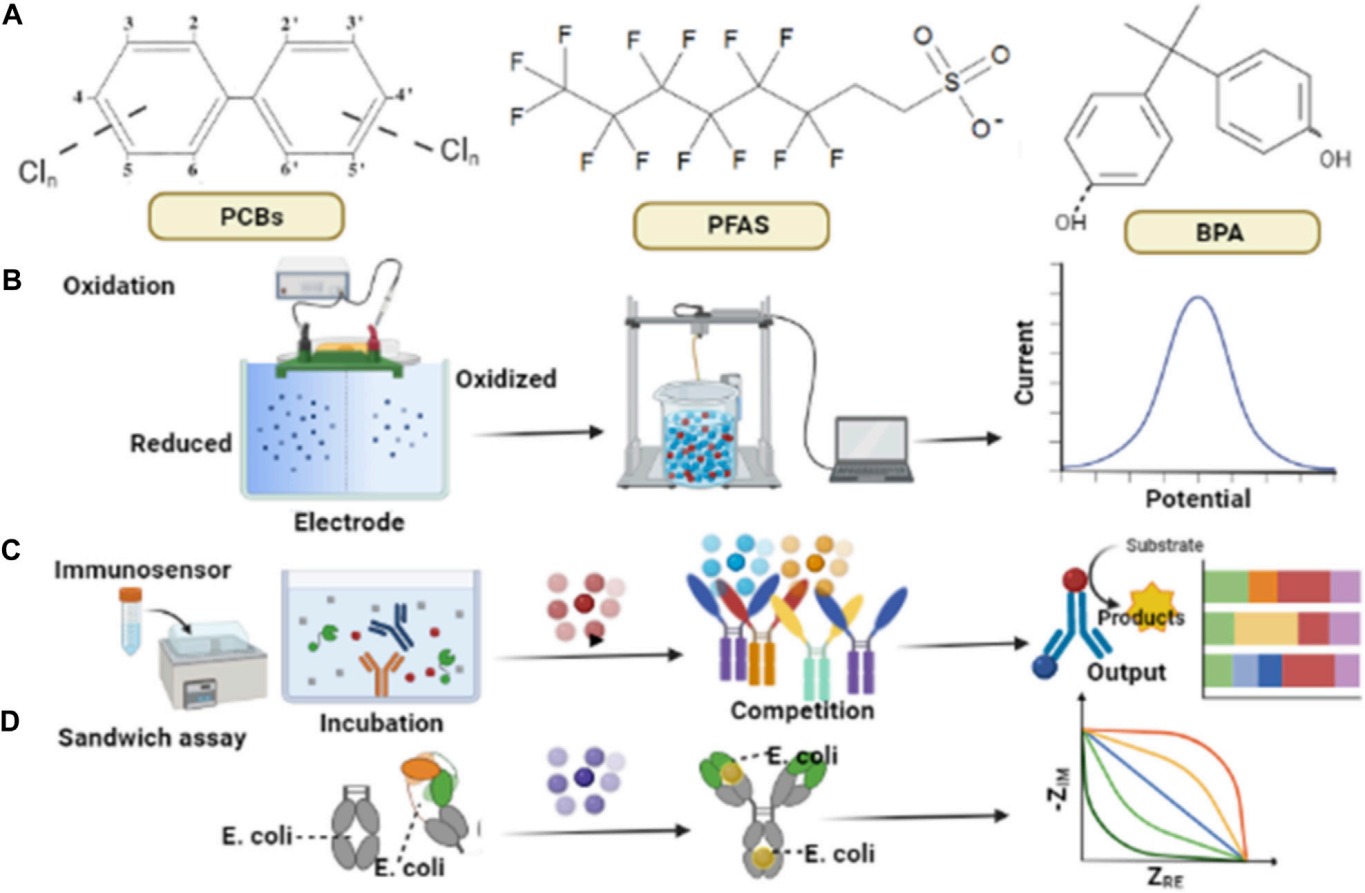
Schematic diagram representing well-developed electrochemical approaches for EDCs monitoring **(A)** Common EDCs with chemical structures **(B)** BPA can be detected by the electro-chemical oxidation methods to find out the presence of its phenolic groups, **(C)** Competition-based immunoassay assay for PCB sensing, enzymes connect with circular dots of different colors which is pre link to the surface, as a result of this connection the electrochemical signals are being generated depending upon PCB amounts in the solution, **(D)** An electrochemical sandwich assay, functioning on the binding of E-coli to an electrode is being sketched to monitor EDCs.

EDCs such BPA and phthalates, due to their electroactive nature, can be detected directly by adopting suitable electrochemical sensing approaches as illustrated in Figure 1. Some common EDCs along with their chemical structures are depicted in Figure 1A. Figure 1B illustrates the direct detection of electroactive species. Figure 1C elaborates on the sensing of EDCs such as polychlorinated biphenyls (PCBs) and PFAS that need an adjunct electro-active species to be measured. These non-electrostatic targets can be identified after interaction with electro-active substances. Figure 1D, shows the detection of binding functionalities of microorganisms *via* electrochemical sandwich assay (Ranaweera et al., 2019; Sofen and Furst, 2019). An electrochemical assay was constructed by expressing estrogen receptors on the surface of *Escherichia coli* (*E. Coli*) for monitoring various EDCs by signal variants from impedance measurements (Furst et al., 2017).

Recent electrochemical sensing systems have also used nanomaterials to enhance the selectivity and sensitivity of the systems (Guo and Irudayaraj, 2011; Stefan-van Staden et al., 2019). The incorporation of nanomaterials such as carbon nanotubes, graphene, and other metal-based nanomaterials onto electrodes provides more selective and sensitive results by improving signal-to-noise ratios on the sensing electrode (Qu et al., 2019; Sofen and Furst, 2019; Ahmad et al., 2022).

#### 2.1.1 Detection of bisphenol A

BPA is an ideal candidate for direct detection due to its electroactive nature, undergoing two-electron, two-proton oxidation, and detection can be amplified with combinations of enzyme reactivity and signal enhancement. Liu et al. (2019) developed an electrochemical biosensor for the monitoring of BPA with the help of a tyrosinase enzyme. In this method, biochar nanoparticles (BCNPs) are synthesized from sugarcane sources by applying the Nafion embedding method, which is being used as a transducer and signal enhancer. The presence of highly conductive BCNPs not only increased the sensor signals but also decreased the impedance and reduction potential, which ultimately improved the output from amperometric sensing. By utilizing this sensor, as little as 3.18 nM of BPA can be detected with a linear range between 0.02 and 10 µM. One important aspect of implementing these technologies is the ability to commercialize testing platforms. These BNCP-enzyme platforms show 86.9% signal retention after 1 month of sensor storage, which confirms the stability of BCNPs/Tyr complex. BNCP-enzyme coupled biosensors provide easy electrode preparation, non-complicated operation, and better performance for the detection of BPA from water samples (Liu et al., 2019).

#### 2.1.2 Detection of atrazine

Atrazine is an herbicide commonly found in ground water and surface runoff (Bachetti et al., 2021; Urseler et al., 2022). Supraja and co-researchers created a real-time sensor for atrazine using an electrospun SnO_2_ nanofiber platform. Using label-free transductions on this platform, atrazine can be detected at 0.9 zM with a linear range of 1.0 zM to 1.0 μM. This system exhibits excellent interference resistance as spiked ground and mineral water samples were successfully tested for atrazine (Supraja et al., 2019). Zahran et al. (2020), used silver nanoparticles (AgNPs) synthesized by the direct reduction technique for the electrochemical sensing of atrazine. Signal enhancement was facilitated with dissolved organic matter (DOM) interacting with AgNPs on the surface of the glassy carbon electrode (GC), leading to the formation of a DOM/AgNP/GC composite. After optimizing the composite manufacture, atrazine was detected in the nM range in stream water samples with this method (Zahran et al., 2020).

#### 2.1.3 Monitoring of phthalate

Dibutyl phthalate (DBP) is used during the manufacture of flexible plastics, as an insect repellent, and as a solvent for oil and resins in several products (Batool et al., 2022; Sun et al., 2022). Bolat et al. (2019), developed a sensor using molecular imprinting for the targeted detection of DBP. An integrated approach using a conducting polymer, polypyrrole (PPY), molecularly imprinted by polymerization in the presence of DBP on a graphite electrode was used. Electrochemical impedance spectroscopy (EIS) is used to detect the interaction of solution DBP with the molecularly imprinted sensor. After sensor optimization, the device can detect 4.5 nM DBP with a linear response range of 0.01–1.0 μM. This sensor provides a rapid, sensitive, economical, disposable, and easy-to-use platform for the detection of DBP (Bolat et al., 2019). Also to detect DBP, a biosensing platform based on a competitive binding assay similar to enzyme-linked immunosorbent assay (ELISA) was developed using gold nanoparticles (AuNPs) for signal amplification. The amplification results from the enlargement of AuNPs *via* catalytic precipitation. Under optimized conditions, the LOD of this system at 7.0 nM is 10 times lower than conventional ELISA. Detection efficiency remains stable at a wide range of pH (6–9) and ionic content (Na^+^8%, Ca^+2^ 4%; w/v) as well as in matrices of pond water and river water. The overall performance of this immunosensor indicates the potential to be developed into a rapid and low-cost sensor for monitoring DBP in water samples (Liang et al., 2017).

#### 2.1.4 Sensing of perchlorates

Perchlorate contamination is primarily associated with military installations due to its use as a strong oxidant in explosives but is also found in food packaging (Feng et al., 2022). It has been found in both drinking water and food (Tian et al., 2020). Hassan and his colleagues developed an electrochemical sensor for perchlorate ions using a solid-contact ion-selective electrode (SC-ISE). The use of single-walled carbon nanotubes as a solid-contact material provides a high double layer capacitance as a result of the high surface area of the nanomaterial. The sensor showed enhanced selectivity toward perchlorate ions with a broad linear range spanning 1 μM–10 mM concentrations and a detection limit under 200 nM. The system showed quick response time, high sensitivity, and accuracy. These types of sensors could be used for the perchlorate determination in a flow system for continuous monitoring (Hassan et al., 2019).

#### 2.1.5 Detection of poly-fluoroalkyl substances (PFAS)

Perfluorooctane sulfonate (PFOS) was widely used in stain-resistant fabrics, fire-fighting foams, food packaging, and as a surfactant in industrial processes, and PFOS can also be generated in the environment after release and transformation of precursors (Kancharla et al., 2022; Nilsson et al., 2022). Although no longer produced in the United States, PFOS remains an environmental contaminant and is still being produced and used in products internationally (Pontius, 2019). Li et al., have developed a low-cost, disposable photoelectrochemical (PEC) strip to monitor the PFOS precursor perfluorooctane sulfonyl fluoride (PFOSF). PFOSF has been recalcitrant to measurement without chemical derivatization. The PEC strips are fabricated *via* a facile one-step electrodeposition method, coated with bismuth oxyiodide (BiOI) nanoparticles, and targeted to PFOSF detection with molecular imprinted polymers grafted to the electrode. The resulting sensors have a linear range of detection of 0.1–1 μM and are capable of detecting PFOSF in spiked river and lake water (Li et al., 2018c). An electrochemical biosensor for PFOS based on the inhibition of the biocatalytic process in enzymatic biofuel cells (BFC) was established. This single-compartment BFC was equipped with multi-walled carbon nanohorns (MWHNs). Glassy carbon electrodes (GCE) acts as a substrate for bio-anode as well as for bio-cathode and glutamic dehydrogenase (GLDH) behaved as biocatalyst for the bio-anode. It had previously been noted that PFOS altered the glutamate mediated current in rat hippocampal cells (Liao et al., 2009). In general, the detection levels from the biosensors were not currently on par with the conventional methods but future improvements are expected. Sensitivity targets to monitor the minimum reporting levels (usually in the 2–4 ppt range) and lifetime health advisory levels (0.004 ppt for PFOA, 0.02 ppt for PFOS, 10 ppt for GenX, and 2,000 ppt for PFOS) are mandated by the EPA in their 2022 reports (Hogue, 2022).

### 2.2 Colorimetric sensors for the detection of EDCs

**FIGURE 2:**
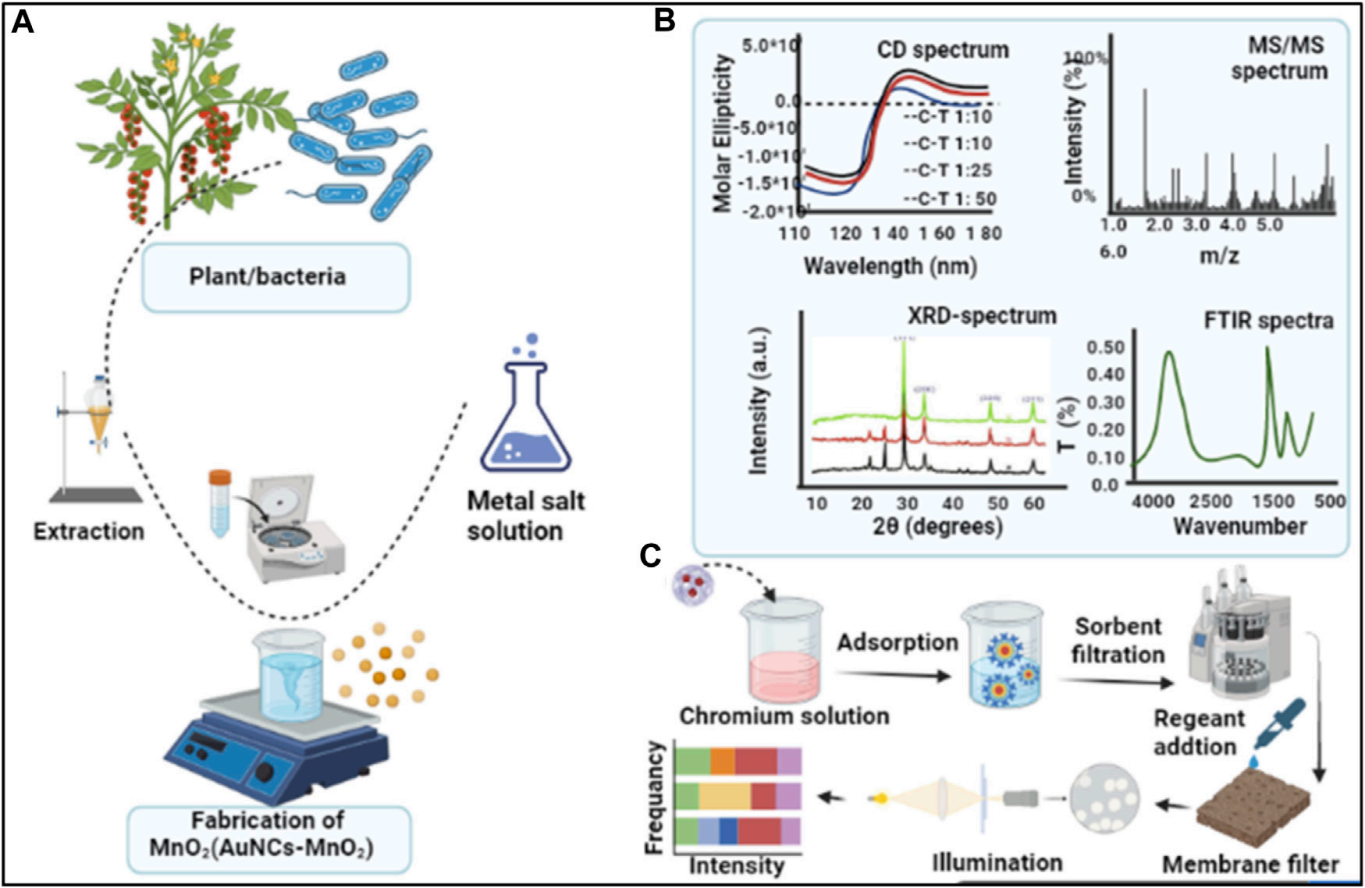
Steps for the colorimetric monitoring of analytes **(A)** Synthesis of MnO_2_ (AuNCs-MnO_2_) nanoparticle from plant/bacteria source, **(B)** fabricated nanoparticles are characterized with different techniques, **(C)** colorimetric detection of an analyte by adapting fluorescence and colorimetric signal variation.

Colorimetric-based sensing systems depend on the color variations in the reaction mixture due to the presence of a specific analyte. The presence of an analyte produces a reaction with the sensing materials, which causes visual color variation (Rodrigues et al., 2021). These sensor platforms provide advantages over existing lab-based methods such as ease of use, quick response, and the ability to obtain test results without any accessory equipment or access to power sources. The quick and distinct color change of the reaction enables users to interpret the positive results qualitatively with the naked eye or quantitatively with optical sensors. The colorimetric assay has proven to be a powerful analytical approach for EDCs, owing to its convenience and simplicity (Shah et al., 2013; Che Sulaiman et al., 2020). An example schematic for novel colorimetric sensing comprising of synthesis steps, characterization, and application (AuNCs-MnO_2_) for monitoring target analytes is outlined in Figure 2. The fabrication of MnO_2_ (AuNCs-MnO_2_) NPs from bacteria/green source is depicted in Figure 2A, and their characterization with various techniques in Figure 2B, while Figure 2C illustrates sample analysis and signal detection by colorimetry.

#### 2.2.1 Colorimetric sensor for detection of perchlorate

The physio-chemical nature of gold nanoparticles is being exploited for onsite visual detection of perchlorate. A reliable nanoparticle-based analytical method for perchlorate detection was proposed with the help of methylene blue (MB) and negatively charged gold nanoparticles. The presence of MB gives an overall positive charge to the AuNPs-MB nanocomposite, which enables the surface to electrostatically attract negatively charged perchlorate which leads to the ion-pair formation between the MB and perchlorate anion. This ion pair formation causes agglomeration in the AuNPs, which is confirmed by the localized SPR absorption band. The LOD of this system was found to be 240 mM and the limit of quantification (LOQ) was observed as 830 mM. The efficacy of this system for percolate ions is being investigated statistically for different interfering common ions (Keskin et al., 2020).

#### 2.2.2 Colorimetric sensor for the detection of pesticides

In a study by Yan et al. (2019), a nanocomposite based on gold nanoclusters and MnO_2_ (AuNCs-MnO_2_) was used to detect carbamate pesticides by adopting fluorescence and colorimetric signal variations. MnO_2_ quenched the fluorescence of AuNCs, which cause a decrease in fluorescence as well as a color change. The presence of acetylcholinesterase (AChE), its substrate, and choline oxidase lead to the generation of H_2_O_2_ due to enzymatic hydrolysis resulting in the decomposition of MnO_2_, affecting the fluorescence from AuNCs. But when carbamate is in the system, its blockage of AChE leads to the reduction of the enzymatic reaction, hence a drop in H_2_O_2_ generation, which eventually results in the blockage of MnO_2_ breakage and increase in quenching of AuNCs fluorescence (Yan et al., 2019).

A simple, easy-to-use dual-sensing system for organophosphate (OP) pesticides using adenosine triphosphate (ATP) and rhodamine B-modified gold nanoparticles (RB-AuNPs) has been successfully developed (Li et al., 2018b). This sensing system is based on the aggregation of AuNPs due to ligand replacement, which is caused by the presence of OP. Ethoprophos, an OP ester, can be detected by the variations in colorimetric and fluorescence signals grom the RB-AuNPs. The linear range for this detection system was 4.0–15.0 µM, and the LOD of this system was as low as 37.0 nM. This cost-effective, sensitive and reliable sensing platform can be applied to real-time sensing of ethoprophos in water samples (Li et al., 2018b).

A colorimetric method for the monitoring of OP is being developed by the aggregation of lipoic acid-capped AuNPs and subsequent color change due to AChE/ATCh (acetylthiocholine) reaction. In this scheme, the enzymatic product of AChE, cationic thiocholine (TCh), causes the aggregation of AuNPs which leads to a change in color from red to blue. The presence of OP in this system causes the blockage of AChE, which ultimately reduces TCh generation, preventing aggregation of AuNPs and the absence of a red-shift in the system. This detection system was applied to detect OP from fruit samples (Sun et al., 2011).

### 2.3 Optical sensors for the detection of EDCs

Optical sensors are highly attractive devices that use light to detect and measure changes in a physical entity, such as position, motion, light intensity, wavelength, and temperature. These sensors have been applied to detect very low concentrations of EDCs in various samples, such as food, beverages, blood, urine, soil, crops, industrial effluents, wastewater, and surface water (rivers, lakes, and oceans). Detection of EDCs in blood and urine helps in the diagnosis of EDC-related disorders and monitoring treatment progress. These chemicals were monitored in soil and crop samples by employing optical sensors to identify the sources of contamination and to undertake measures to reduce their impact on crop yields and soil fertility. Monitoring industrial effluents and wastewater can help in the identification of sources of contamination whereby measures can be undertaken to reduce their release into the environment. Optical sensors can be used to assess the extent of contamination and to take measures to mitigate its effects.

#### 2.3.1 Optical sensors for the detection of atrazine

A fluorescent-based photo-stable detection system of atrazine was developed by Mohapatra et al. (2018), with the help of a nitrogen-doped carbon quantum dot. The presence of atrazine induces the fluorescence system to luminesce at increasing intensity depending on the concentration of atrazine. The hydrogen bonding interaction between the atrazine and amino groups on the surface of the quantum dots makes the sensing system selective and highly sensitive to atrazine. With this approach, as low as 3.0 pM of atrazine could be detected in the broad linear range of 5.0 pM–7.0 nM. This system is being applied to not only the real-time detection of atrazine in agricultural samples but also to detect atrazine in bacterial cell lines were the nitrogen-doped carbon quantum dots show good cell permeability and emission intensity in bacterial cell lines (Mohapatra et al., 2018).

**FIGURE 3:**
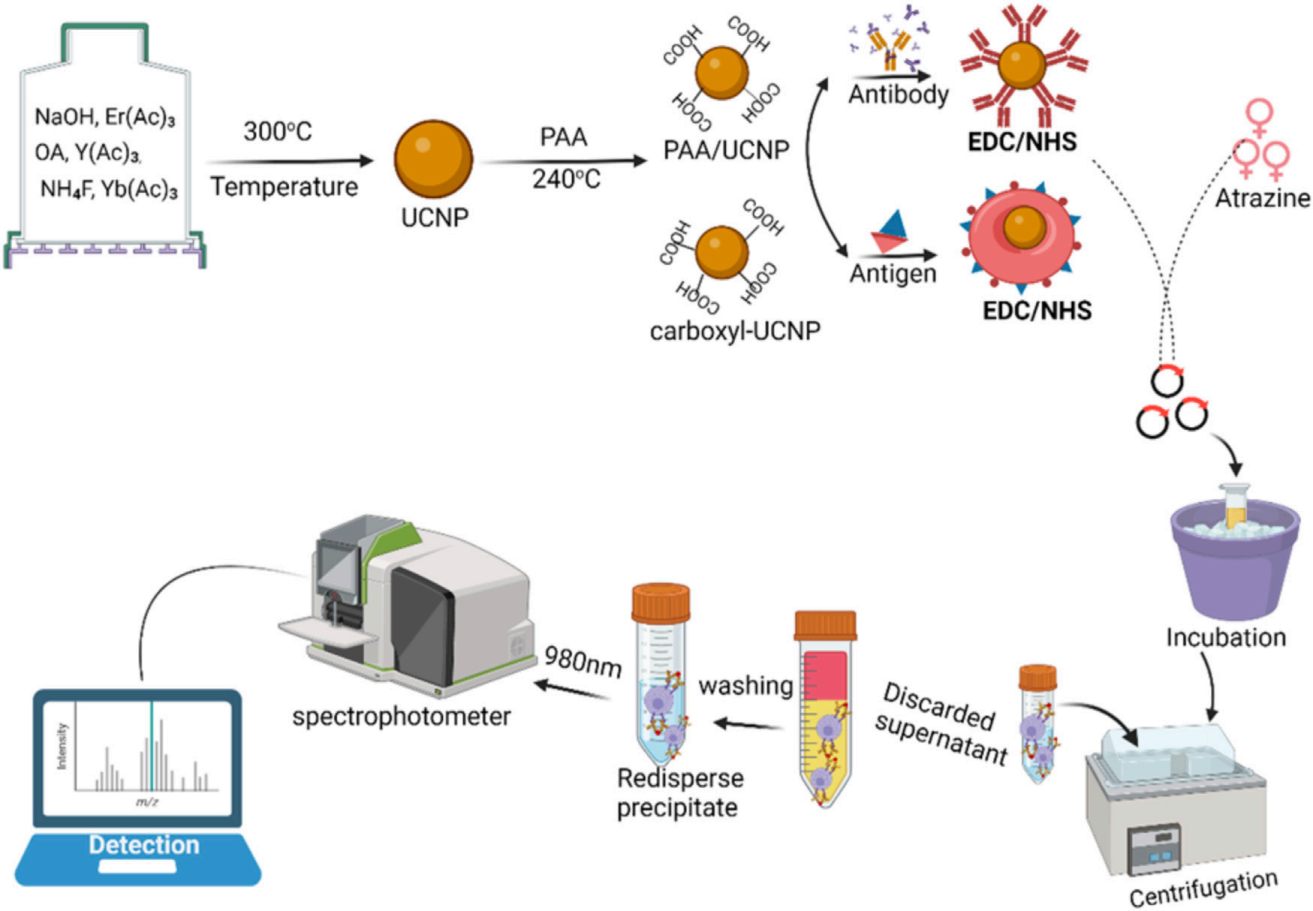
An illustration of probe fabrication and analysis based on immunofluorescence (competition assay for atrazine detection).

The detection principle of a novel fluorescence immunoassay for atrazine is shown in Figure 3. Herein, the probe [comprising of the antigen-polystyrene magnetic microspheres (PMMs) complex] can be used to capture the target [coating antibody-upconversion nanoparticles (UCNPs) blend] to form an immune conjugate (antibody-UCNPs-atrazine-PMMs) and detected with a spectrometer. The use of an association between the atrazine and the fluorescence signal allows for quantitative sensing of atrazine in the sample (Sheng et al., 2019).

#### 2.3.2 Optical sensors for detection of aromatic ring compounds

Li et al. (2021), adopted a fluorescence-based method for the ultrasensitive detection of BPA in water samples with the application of up-conversion nanoparticles (UCNPs) and tetramethylrhodamine. The use of UCNPs not only enhanced the sensitivity of the system, but the luminous efficiency also increased. A simple pretreatment for the water sample measured makes this method time-saving and easy to adopt (Li Q et al., 2019).

An assay for detecting BPA using a material created by compounding magnetic Fe_3_O_4_ onto the surface of oxidized graphene to make magnetic oxidation graphene (MGO) was developed. The magnetic material is a strong quenching agent. Fluorescently labeled BPA-targeted single-stranded DNA (ssDNA) aptamer sequences bind to the surface in a quenched state. The presence of free BPA releases the aptamer and restores the fluorescence. Magnetic separation of the MGO material after release of fluorescent aptamer provides a sensitive indicator of the presence of BPA. The LOD of this system was 0.071 ng/mL and a linear range of 0.2–10 ng/mL. The specificity of the aptamer for BPA and the use of fluorescence proves to be highly sensitive and selective (Hu et al., 2017). Using a similar targeted binding strategy, Inuyama and co-researchers capitalized targeted dioxin-binding peptide in a competitive binding assay with fluorescence readout. The fluorescent compound *N*-NBD-3-(3′, 4′-dichlorophenoxy)-1-propylamine binds to the dioxin-targeted peptide which is conjugated to the surface of a bead. The fluorescence of the beads decreases as increasing concentrations of dioxin displace the fluorescent molecule from the bead. The fluorescence intensity decreases with the increase in the concentration of dioxin. With optimized bead conjugations an LOD of 0.5 nM dioxin is achieved. This system is robust enough to identify soil samples that have a higher concentration of dioxin than standards (Inuyama et al., 2007).

A Surface Plasmon resonance (SPR) based sensing assay was developed by capitalizing on functionalized gold nanoparticles to enhance sensor response. By adopting this strategy BPA was successfully detected in the concentrations ranging from pM to mM with a detection limit of 22.7 pM (Xue et al., 2019). Another sensing system was designed for monitoring BPA based on molecular imprinted photonic crystals. The high specificity and selectivity of the developed sensing system were associated with the interactions of binding sites of nano-cavities with BPA (Griffete et al., 2011). An efficient, regenerative two dimensional, dually cross-linked photonic crystal for colorimetric and florescent-based sensing system for BPA monitoring was developed. The detection limit of this cost-effective and rapid system was 4.38 nM (Du et al., 2023).

Another SPR-based sensing system was developed for monitoring of triclosan in wastewater. The sensor was modified to enhance its functionality by the incorporation of allylmercaptane-modified gold SPR chip and imprinted poly(2-hydroxyethyl methacrylate–methacryloylamidoglutamic acid) nanofilm. The linearity range and detection limit of this sensor were 0.173–3.45 nM and 0.058 nM, respectively (Atar et al., 2015). A modified quartz crystal microbalance (QCM) was established to inspect four EDCs including BPA, oestrone, oestradiol, and sulfamethoxazole, by employing an adsorption mechanism *via* a zeolite filter in real-time in water samples. Observed results correlate with the models of pseudo-first-order kinetic and Sips isotherms. Adsorption efficiency was influenced by the molecular structure and polarity of these analytes, but the variations in pH and ionic strength of the sample did not impact adsorption efficiency (Li et al., 2021).

#### 2.3.3 Monitoring of phthalate esters by optical sensing systems

Chapartegui-Arias et al. (2019), established a zeolitic imidazole framework (ZIF) to measure phthalate esters (PAEs) in solution by an optical sensing protocol. In this method, the fluorescence emission intensity of aminopyrine conjugated to the ZIF changed due to the presence of Phthalate. Aminopyrene is covalently bound to the zeolitic imidazolate framework (ZIF-8) and the addition of a butylamine as a modulating agent during synthesis resulted in nanoparticles of ZIF-8. The quenching of the fluorescence is observed when PAEs are present and interact with the material by their aromatic structures. The ability to capture transient changes in fluorescence intensity without expensive and time-consuming regeneration steps show the possibility of a cheap, fast, and easy-to-use PAEs detection system. With some initial pretreatment of industrial wastewater, this detection system can be used for quick and reliable PAEs sensing with the LOD range from 0.064–0.19 mM (Chapartegui-Arias et al., 2019).

A novel fluorescence-based molecularly imprinted polymer (MIP) synthesized by precipitation polymerization for the monitoring of dibutyl phthalate (DBP) has been described. Mn-doped ZnS quantum dots (QDs), which anchor a MIPs layer on the SiO_2_ nanoparticles (SiO_2_@QDs@MIPs), were synthesized. Acrylamide was used as a functional monomer and ethyl glycol dimethacrylate as the cross-linker in the presence of DBP to create the MIP layer on the surface of the SiO_2_ nanoparticles. The sensor can selectively and sensitively recognize DBP resulting in a decreasing fluorescence intensity of the particles with increasing binding of DBP. In optimized conditions, the fluorescence signal changes linearly with concentration of DBP. The SiO_2_@QDs@MIPs was applied to tap water analysis, and on average, a 97.80% recovery rate of DBP was observed with a relative standard deviation (RSD) of less than 3.25%. This new method provides excellent, quick, responsive, stable, and applied alternatives to sophisticated analytical tools for the onsite detection of DBP in the linear range from 5 to 50 µM (Zhou et al., 2017).

An immunofluorescence plate assay was developed by (Zhang et al., 2006). A polyclonal antiserum was generated in rabbits targeting DBP. A very low cross-reactivity rate (10%) with similar phthalate compounds suggests polyclonal antiserum can increase sensitivity with an acceptable loss of specificity. The resulting antibody-coated plate format was used for the detection of dibutyl phthalate (DBP) *via* fluorescence immunoassay. With the help of this assay, water samples from different sources such as river water, tap water, and drinking water can be tested for the DBP with simplicity, sensitivity, and reliability (Zhang et al., 2006).

#### 2.3.4 Optical sensor for detection of pesticides

A novel, indirect strategy for detecting the insecticide parathion-methyl (PM) based on the measurement of its hydrolysate, p-nitrophenol, was established by Yan et al. (2015). The fluorescence of CdTe quantum dots (QDs) assembled with positively charged cetyltrimethylammonium bromide (CTAB) is quenched when the alkyl chain of CTAB interacts with the aromatic ring of p-nitrophenol. The compound p-nitrophenol is produced by the hydrolysis of PM by organophosphorus hydrolase (OPH) and is subsequently involved in the electron transfer from CdTe QDs/CTAB. The long alkyl chain of CTAB possesses a positive charge that is an absorbent for p-nitrophenol due to hydrophobic interactions. The presence of PM is indirectly assessed from the fluorescence variation of CdTe QDs/CTAB by this electron transfer approach at a detection limit of 0.062 µM (Yan et al., 2015). For real-time, precise, and accurate detection of a herbicide (2,4-dichlorophenoxyacetic acid), molecularly imprinted QCM and SPR systems were proposed. Polymeric surfaces were designed by exploiting the molecular imprinting procedure to develop sensors with high sensitivity. Observed LOD of the QCM and SPR sensors was 0.091 and 0.11 nM respectively (Cakir et al., 2019).

#### 2.3.5 Sensing of PFAS by adopting optical sensors

A spectrophotometric turn-on method was devised for the detection of PFOS/PFOA in environmental water samples using an erythrosine B (EB) and CTAB fluorescence system. The fluorescence of EB in the presence of a large excess of CTAB results in both a quenching and a fluorescence redshift of 11 nm. The addition of PFOS/PFOA induces enhanced fluorescence intensity at the new redshifted wavelength. It is found that the presence of PFOS/PFOA enhanced the fluorescence in proportion to the concentration of these toxins. The LOD for PFOS was 12.8 nM and for PFOA it was 11.8 nM. The established method is a natural, simple, quick, economical, and sensitive way to monitor the PFOS/PFOA in the water sample (Cheng et al., 2018). However, the detection limits achieved may not be sufficient to detect trace levels of PFAS. Using a similar strategy (Cheng et al., 2019), further discovered that in a sensing setup, PFOS can be visually detected from the aqueous samples with the help of carbon dots (CDs) and berberine chloride hydrate (BH). The presence of BH decreases the fluorescence of CDs in the medium of the Britton-Robinson (BR) buffer solution (pH 6.09). The addition of PFOS in the system recovers the fluorescence slightly at 488 nm but greatly enhances fluorescence at 533 nm. Interestingly this effect was not observed with other perfluorinated compounds of this family. Visual color variations from blue to light yellow were easily perceived in the presence of PFOS. The enhanced intensity responses at 533 nm are linear in the range of 0.22–50.0 µM PFOS concentrations with a LOD at 21.7 nM (Cheng et al., 2019).

### 2.4 Other enhanced approaches for the detection of EDCs

Recognition and detection of EDCs with the help of antibodies, aptamers, and hormones integrated into a sensing setup provide sensitive and selective detection. This setup also provides and enhances the knowledge of the interaction of EDCs with living systems (Sofen and Furst, 2019). Aptamers are artificial manmade nucleic acid ligands that can be produced to bind to any target molecules including proteins, amino acids, and drugs (O'Sullivan, 2002).

#### 2.4.1 Aptamer-based detection approaches for the monitoring of EDCs

An aptamer-based electrochemical sensor for atrazine (ATZ) was developed by Fan et al. (2019). Nickel hexacyanoferrate nanoparticles (NiHCF NPs) and electrochemically reduced graphene oxide (ERGO) were applied to modify a glassy carbon electrode (GCE) surface. AuNPs were deposited on the electrode to facilitate the anchoring of atrazine-targeted aptamer. When atrazine is introduced into the system, it forms a complex with the aptamer. This ATZ-aptamer complex leads to a decrease in the electrochemical signal due to the hindrance of electron transfer. The linear curve with this sensing system was in the range of 0.25–250 pM with the LOD 0.1 pM. This method could apply to the detection of ATZ in water and soil samples (Fan et al., 2019). In another study, Jia et al. (2020), took a previously developed 63-mer aptamer targeting BPA and rationally truncated the sequence into two shorter, higher affinity aptamers of 38-mer and 12-mer length. A comparison of the sensing system with original and truncated aptamers was performed. In comparison to the parent aptamer, the LOD of the 38-mer was 265-fold higher the original aptamter and the LOD of the 12-mer was 14-fold higher. In the same way, the selectivity of truncated aptamers was found to be much higher than the original aptamer. The sensor shows the potential for sequential development and optimization of aptamers for EDC targeted sensors. The developed sensor was used to detect BPA in milk, orange juice, and water showing potential for use in food and environmental samples (Jia et al., 2020).

A similar strategy of optimizing aptamer binding was used to develop a sensor for the detection of the plasticizer di-2-ethylhexyl phthalate (DEHP). A Quantum dot aptasensor (QD-aptasensor) with a portable analyzer was developed. Three truncated aptamers of 45-, 28-, and 22-mer were taken from a previously identified 60-mer aptamer targeting DEHP along with three unique DNA probes. Of all the different combinations, the 22-mer aptamer with 12mer DNA probe provides excellent sensitivity and selectivity even in the presence of phthalate analogs and a linear signal response to increasing concentrations of DEHP. The selected aptamer-probe combination is being tested further to make the sensing system more amenable to practical use (Lim et al., 2018). Using a 24-mer aptamer coupled to gold nanoparticles (AuNP-aptamer), Lee and co-researchers created an easy and quick colorimetric assay for the detection of BPA. In the addition of BPA, the AuNP-aptamer undergoes electrolytic induced aggregation, resulting in a visual color shift from red to blue in presence of part per billion concentrations of BPA. With the help of this method, BPA can be detected at concentrations as low as 4.0 pM. Additionally, the performance of this system is comparable to analytical methods such as chromatography and ELISA (Lee et al., 2019).

#### 2.4.2 Enzyme-based monitoring systems for the detection of OP

An enzyme-based detection system for the organophosphate pesticides dichlorvos and methyl-paraoxon was developed by (Zhang et al., 2016) by exploiting the toxic, acetylcholinesterase (AChE) inhibition caused by OPs. The system uses polyacrylic acid-coated cerium oxide nanoparticles (PAA-CeO_2_) to oxidize the chromogenic substrate tetramethylbenzidine (TMB) to create a blue colored solution. The enzymatic activity of AChE in the system reduces acetylthiocholine to thiocholine (TCh). The resulting TCh inhibits the oxidation of TMB, resulting in a clear color. The addition of OPs inhibits the creation of TCh, allowing more TMB to be oxidized and create a blue color. The system can detect dichlorvos as low as 8.62 ppb and methyl-paraoxon at 26.73 ppb. This colorimetric approach is a potential tool for rapid screening for the presence of OPs (Zhang et al., 2016).

#### 2.4.3 Detection of PFAS with SPR sensing approaches

A surface plasmon resonance (SPR) optical fiber biosensor was developed by Cennamo et al. (2018b), to detect perfluorooctanoate (PFOA) and Perfluorooctane sulfonate (PFOS) compounds. Polyclonal antibodies were produced in rabbits and the resulting serum was enriched for target specificity using PFOA-EAH sepharose columns. The resulting antibodies show similar affinity to both PFOA and PFOS, likely due to the method of binding PFOA to the sepharose column. This system has been applied for the sensing of PFOA/PFOS in seawater. This sensing platform can detect concentrations of PFOA/PFOS as low as 0.221 ppb, lower than the maximum residue limit for PFOA/PFOS set by European Union regulations (Cennamo et al., 2018b). A conceptually similar approach was adopted to create SPR optical fiber biosensors using Molecularly Imprinted Polymer (MIP) materials instead of antibodies. This system was selective toward a range of per-and polyfluoroalkyl substances (PFAS) with carbon backbones in the C4-C11 range. The sensor detected these compounds with LOD of 0.13–0.15 ppb. Its performance was comparable to the antibody-based SPR-POF system but possessed the advantages of cost-effectiveness, reproducibility, and increased stability with natural, environmental solutions (Cennamo et al., 2018a).

### 2.5 Microbial sensing system for detection of EDCs

**FIGURE 4:**
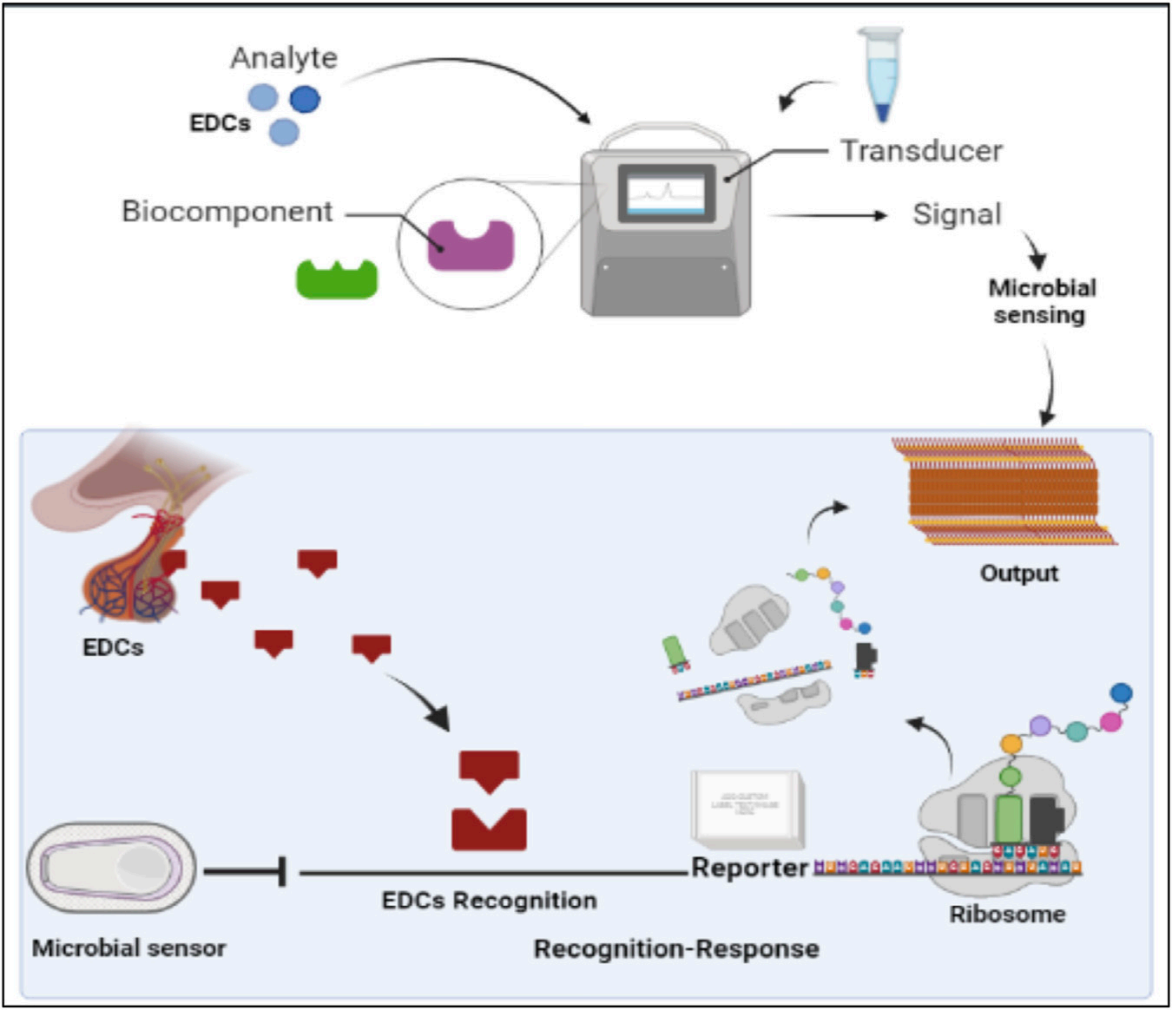
The illustration of cell free microbial sensors for monitoring EDCs. Cell free microbial sensors were used to detect EDCs. The downstream reporter gene is activated for transcription and then translated for reporter protein production in a cell free system.

Various microbial sensors have been reported for the monitoring of EDCs. Generally, this class of sensors consists of a microbe as both a detector and a transducing agent as elaborated in Figure 4. These systems build on detection and signaling pathways that have previously evolved in organisms to improve survival potential in the environment. The specificity and sensitivity of these sensors to EDCs has been enhanced by the genetic modification and regulation of gene expressions of these microbes. As a result, the efficiency and sensitivity of these systems has increased to compete with standard immunosensor technology (Bilal and Iqbal, 2019). These sensors provide some advantages over standard sensors such as robust response in a range of measurement conditions, extended lifetime, and low cost. One disadvantage of these systems is the need for a prolonged response time compared to other sensing systems.

An *E. coli*-based lux operon biosensor for the efficient testing of perchlorate was developed. The lux biosensor strains tested generate a bioluminescence in response to reactive oxygen species. The presence of ammonium perchlorate (AP) in the media causes the generation of hydrogen peroxide and superoxide anion, which causes oxidative stress in the sensor cells. During the design of lux biosensors, hybrid plasmids are fused with bacterial luciferase lux genes. The concentration of AP at 5.0 and 50 mM induces 5 and 10 times higher luminescence respectively in bacterial cells relative to untreated cells (Balabanov et al., 2017). An array of M13 bacteriophage is being exploited to develop a colorimetric sensor for the detection and categorization of EDCs, including benzene, phthalates, and polychlorinated biphenyls (PCBs). Structurally and genetically modified M13 bacteriophages are being used for the biomimetic sensor, which changes the color patterns depending on the compound. This promising, portable, and easy-to-use sensing system could be applied for the monitoring of food and pharmaceutical samples (Moon et al., 2016).

## 3 Challenges and limitations of sensing EDCs systems

Various types of sensing techniques including electrochemical, colorimetric, and optical sensing technologies have been developed for monitoring EDCs in various environments, such as water, food, and consumer products. However, these technologies also face several challenges and limitations. As such, these sensors have varying levels of sensitivity to the different EDCs, and poses a challenge for detection with sufficient sensitivity. These sensors may also face challenges in distinguishing between different EDCs or between EDCs and other chemicals that may be present in the sample. The presence of other chemicals or compounds in the sample may interfere with the functioning of the sensor, leading to false positives or false negatives. In some cases, sample preparation may be required before analysis, which can add to the complexity of detection. In addition, the performance of these sensors may be affected by environmental factors such as temperature, humidity, and pH, which can lead to variations in their accuracy and reliability. The presence of different types of matrices (e.g., soil, water, food) can also affect the performance of these monitoring systems since the properties of the matrix may interfere with the functioning of the sensor or affect the detection signal. Overall, while sensing technologies offer promising solutions for the monitoring of EDCs, there are still several challenges and limitations that need to be addressed to improve their accuracy, sensitivity, and practicality for use in real-world settings.

## 4 New trends and future prospects

Advanced sensing techniques hold significant promise in the monitoring of EDCs in the environment. New nanomaterial-based sensors can provide increased sensitivity to detect EDCs at very low concentrations. This can lead to more accurate and reliable monitoring even in complex matrices. Sensing techniques based on molecularly imprinted polymers (MIPs), aptamers, or other biomimetic materials have the potential to be highly specific to the target EDC. This can minimize interference from other compounds in the sample and reduce the occurrence of false positives and has the potential for rapid response. This can be especially important in industrial or agricultural settings where there is a higher risk of EDC contamination. Sensor technologies can also allow for remote monitoring of EDCs using wireless or internet of things (IoT) technologies. Advance techniques can provide timely data to environmental agencies or other stakeholders, enabling them to take appropriate actions to reduce health hazards. Sensor-based methods can render monitoring EDCs more accessible to a wider audience, including communities and citizen scientists. Platforms can be designed to detect multiple EDCs simultaneously, providing a more comprehensive understanding of environmental contamination. This can enable stakeholders to develop targeted mitigation strategies to reduce exposure to multiple EDCs.

There are several new trends in the development of sensors for the monitoring of EDCs, including integration with fluidics, miniaturization, and multiplexing. The integration of sensors with microfluidics can enable automated and high-throughput sample processing, reducing the time and cost of EDC detection. Microfluidic platforms can also enable precise control of sample flow and reaction conditions, leading to increased sensitivity and specificity of EDC detection. Miniaturization of sensors can enable portable and field-deployable devices. Miniaturized sensors can also reduce the amount of samples and reagents required for detection, making them cost-effective and environmentally friendly. These sensors can also enable *in situ* and real-time monitoring of EDCs in environmental and biological samples. Multiplexed sensors can detect multiple EDCs simultaneously in a single sample, reducing the time and cost of EDC detection. Multiplexed sensors can also enable the detection of complex mixtures of EDCs which often occur in environmental samples. Multi-target sensors can be achieved through the use of arrays of sensors with different sensing elements, or the use of microarrays on lab-on-a-chip platforms. Overall, these trends are enabling the development of advanced sensors for EDC detection that are more sensitive, specific, cost-effective, and portable than traditional methods. These sensors have the potential to revolutionize EDC monitoring and risk assessment, leading to improved public health and environmental protection.

## 5 Conclusion

EDCs are found in a broad spectrum of locations ranging from commercial to residential areas. Many are ubiquitous, posing serious threats to human health. Given the increase in EDCs in the domestic and industrial sectors, improved sensors for point of exposure detection are of the highest need. In the future, advanced EDC sensors for routine monitoring will become imperative to control the deleterious health effects of these compounds. Devices to detect these chemicals at home, in agricultural fields, wastewater treatment units and in manufacturing plants will become the norm. EDC detection is a potentially growing field; non-analytical advanced sensing systems have emerged as a promising means for the detection and monitoring of EDCs due to their high sensitivity, selectivity, cost-effective, portable, rapid response time as well as user-friendly approach. The development of sensors for EDCs has been driven by the need to monitor these chemicals in various settings, including water, air, and food and stringent limits by the regulatory agencies. Biosensors have gained significant attention in the medical industry and are already emerging as an alternative to traditional and conventional methods for the sensing of EDCs. In contrast to traditional analytical sensing techniques, modern sensing methods can provide for the possibility of detecting an array of pollutants and toxic chemicals on site, rapidly and with excellent sensitivity and selectivity.

Despite the significant progress made in the development of sensors for EDCs, challenges remain along with significant opportunities. One of the main challenges is the detection of EDCs in complex environmental sample matrices. Other limitations such as sustainability and utilization under harsh environmental factors such as temperature, alkalinity, pH, salinity, and other adverse conditions can lead to poor results. With advances in sample preparation methods, device durability, and cost effectiveness these limitations can potentially be overcome in the near future. Given the emphasis to reduce pollution in the coming decades, explosive growth in the development and deployment of these state-of-the-art technologies for environmental sensing and detection of EDCs is expected. Overall, the development of sensors for EDCs is an active and growing field. The continued development of sensors for EDCs has the potential to provide valuable insights into the exposure and health effects associated with these chemicals, as well as inform public health policies aimed at reducing health risk due to exposure in a timely manner.
